# Does social cognition change? Evidence after 4 years from the Italian Network for Research on Psychoses

**DOI:** 10.1192/j.eurpsy.2022.2356

**Published:** 2023-01-11

**Authors:** Paola Rocca, Paola Rucci, Cristiana Montemagni, Alessandro Rossi, Alessandro Bertolino, Eugenio Aguglia, Carlo A. Altamura, Mario Amore, Ileana Andriola, Antonello Bellomo, Claudio Brasso, Bernardo Carpiniello, Elisa Del Favero, Liliana Dell’Osso, Fabio Di Fabio, Michele Fabrazzo, Andrea Fagiolini, Giulia Maria Giordano, Carlo Marchesi, Giovanni Martinotti, Palmiero Monteleone, Maurizio Pompili, Rita Roncone, Rodolfo Rossi, Alberto Siracusano, Elena Tenconi, Antonio Vita, Patrizia Zeppegno, Silvana Galderisi, Mario Maj, Italian Network for Research on Psychoses

**Affiliations:** 1 Section of Psychiatry, Department of Neuroscience, University of Turin, Turin, Italy; 2 Department of Biomedical and Neuromotor Sciences, University of Bologna, Bologna, Italy; 3 Section of Psychiatry, Department of Biotechnological and Applied Clinical Sciences, University of L’Aquila, L’Aquila, Italy; 4 Department of Neurological and Psychiatric Sciences, University of Bari, Bari, Italy; 5 Psychiatry Unit, Department of Clinical and Molecular Biomedicine, University of Catania, Catania, Italy; 6 Department of Psychiatry, University of Milan, Milan, Italy; 7 Section of Psychiatry, Department of Neurosciences, Rehabilitation, Ophthalmology, Genetics and Maternal and Child Health, University of Genoa, Genoa, Italy; 8 Psychiatry Unit, Department of Medical Sciences, University of Foggia, Foggia, Italy; 9 Section of Psychiatry, Department of Public Health, Clinical and Molecular Medicine, University of Cagliari, Cagliari, Italy; 10 Section of Psychiatry, Department of Clinical and Experimental Medicine, University of Pisa, Pisa, Italy; 11 Department of Neurology and Psychiatry, Sapienza University of Rome, Rome, Italy; 12 Department of Psychiatry, University of Campania “Luigi Vanvitelli”, Naples, Italy; 13 Department of Molecular Medicine and Clinical Department of Mental Health, University of Siena, Siena, Italy; 14 Psychiatry Unit, Department of Neuroscience, University of Parma, Parma, Italy; 15 Department of Neuroscience and Imaging, G. D’Annunzio University, Chieti, Italy; 16 Department of Medicine, Surgery and Dentistry “Scuola Medica Salernitana”, Section of Neuroscience, University of Salerno, Salerno, Italy; 17 Department of Neurosciences, Mental Health and Sensory Organs, S. Andrea Hospital, Sapienza University of Rome, Rome, Italy; 18 Unit of Psychiatry, Department of Life, Health and Environmental Sciences, University of L’Aquila, L’Aquila, Italy; 19 Psychiatry and Clinical Psychology Unit, Department of Systems Medicine, Tor Vergata University of Rome, Rome, Italy; 20 Psychiatric Clinic, Department of Neurosciences, University of Padua, Padua, Italy; 21 Psychiatric Unit, School of Medicine, University of Brescia, Brescia, Italy; 22 Department of Mental Health, Spedali Civili Hospital, Brescia, Italy; 23 Psychiatric Unit, Department of Translational Medicine, University of Eastern Piedmont, Novara, Italy

**Keywords:** Follow-up, recovery, reliable and clinically significant change (RCSC), schizophrenia, social cognition, theory of mind

## Abstract

**Background:**

Deficits in social cognition (SC) are significantly related to community functioning in schizophrenia (SZ). Few studies investigated longitudinal changes in SC and its impact on recovery. In the present study, we aimed: (a) to estimate the magnitude and clinical significance of SC change in outpatients with stable SZ who were assessed at baseline and after 4 years, (b) to identify predictors of reliable and clinically significant change (RCSC), and (c) to determine whether changes in SC over 4 years predicted patient recovery at follow-up.

**Methods:**

The reliable change index was used to estimate the proportion of true change in SC, not attributable to measurement error. Stepwise multiple logistic regression models were used to identify the predictors of RCSC in a SC domain (The Awareness of Social Inference Test [TASIT]) and the effect of change in TASIT on recovery at follow-up.

**Results:**

In 548 participants, statistically significant improvements were found for the simple and paradoxical sarcasm of TASIT scale, and for the total score of section 2. The reliable change index was 9.8. A cut-off of 45 identified patients showing clinically significant change. Reliable change was achieved by 12.6% and RCSC by 8% of participants. Lower baseline TASIT sect. 2 score predicted reliable improvement on TASIT sect. 2. Improvement in TASIT sect. 2 scores predicted functional recovery, with a 10-point change predicting 40% increase in the probability of recovery.

**Conclusions:**

The RCSC index provides a conservative way to assess the improvement in the ability to grasp sarcasm in SZ, and is associated with recovery.

## Introduction

Social cognition (SC) is a multifaceted construct, that has been defined as “the mental operations that underlie social interactions, including perceiving, interpreting, and generating responses to the intentions, dispositions, and behaviors of others,” thus allowing people to infer meaning from social situations and behaviors and to respond appropriately [1].

The social cognition psychometric evaluation (SCOPE) study [2] identified the following four key domains of SC in schizophrenia (SZ): (a) theory of mind (ToM), that is, the ability to understand the mental states (beliefs, knowledge, and intentions) of other people from their speech, actions, and/or nonverbal behavior, and infer that these may differ from one’s own; (b) emotion perception (both prosodic and facial) or the ability to infer emotional information from facial expressions, vocal inflections, or some combination of these; (c) social perception and knowledge or the ability to identify social roles, societal rules, and social situations; and (d) attributional style or bias, which refers to the process of attaching meaning to behavior, that is, finding reasons for one’s own or another’s behavior an individual’s tendency to attribute the cause of an event to either oneself, others, or the environment.

SC impairments have been documented throughout the disease course in SZ [3], that is, prior to the illness onset [4, 5], early in its course [6, 7], in the established illness, during periods of symptom remission, as well as in clinical high-risk subjects [8–10] and in first degree relatives of patients with SZ [11], thus suggesting a putative genetic vulnerability, rather than a state dependent deficit [12].

Moreover, the bulk of studies has shown a strong link between impairments in both SC and neurocognition (NC) and functional outcomes in SZ [13–16]. Although there are some overlaps, NC and SC are separate constructs, with distinct relationships with functional outcomes [15, 17, 18]. Moreover, mediation results have shown SC is a mediator in the relationship between NC and functional outcomes [19–21]. The proximity of SC to functional outcomes offers support for SC as a primary treatment target for optimal improvement in functioning. A greater understanding of the relationships between NC, SC, and functioning may provide opportunities for targeted recovery-focused interventions in SZ.

Recent meta-analytical evidence has shown that the strength of the association between deficits in SC and deficits in social functioning among individuals with psychotic disorders may be greater than that of the association between NC and social functioning in individuals with psychotic disorders. The quantitative review of Fett and colleagues [14] on 55 studies found small to large mean correlations between NC and SC and functional outcomes, suggesting that SC explains more variance in functional outcomes than NC, with the strongest individual correlations observed between ToM and community functioning (23% variance explained) and attention and vigilance and social skills (15% variance explained), respectively.

In a more recent metanalysis of Halverson [22], that adopted random effects approaches to model summary estimates between NC, SC, and functional domains for the first time, the average variance explained by NC (2–7%) and SC (4–10%) is smaller than individual relationship estimates of the review by Fett et al. [14]. NC and SC appeared to be equally associated with community functioning, SC resulted particularly more strongly associated with social skill and behavior-related outcome (e.g., social behavior and social skills), which in turn may improve community outcomes through better helping networks, while NC may be particularly important for independent living which makes these networks possible [22].

The available evidence is plagued by serious methodological limitations [23], mostly arising from cross-sectional designs, limited sample representativeness and comparability, and measurement equivalence [24–26]. Although several research papers compare recent onset patients with those in a chronic phase, evidence is inconclusive [12], with some studies supporting stability of SC impairment [5, 27], and others showing progressive impairment [28]. Moreover, most of the few longitudinal studies cover short follow-up period (e.g., 6–12 months) [29–31]. The Italian Network for Research on Psychoses (NIRP) study [32] was designed to assess at baseline and 4 years a broad set of symptomatic, cognitive, and functional domains in a large Italian sample of patients with SZ, thereby allowing to capture long-term variations of these domains and their determinants. In the framework of this study, we assessed two SC domains relevant to adaptive social interactions, emotional processing, and ToM, aimed to address some of above-mentioned limitations, and to evaluate the change in SC in patients with SZ at 4 years. In particular, we aimed to: (a) estimate the reliable and clinically significant change (RCSC) of SC scales using data from clinically stable outpatients with SZ who were assessed at baseline and 4 years; (b) to identify predictors of RCSC of SC scales, and (c) to determine whether changes in SC over 4 years predicted patients’ recovery at follow-up.

## Materials and Methods

### Study design and participants

Of 26 Italian university psychiatric clinics or mental health departments initially involved in the NIRP study [26], 24 participated in the follow-up study. All participants fulfilled *DSM-IV* criteria for SZ as ascertained by the Structured Clinical Interview for *DSM-IV*, patient version [33].

The study protocol was approved by the local ethics committees of the participating centers, and recruitment was carried out from March 2016 to December 2017. After receiving a comprehensive explanation of the study procedures and goals, all patients provided written informed consent obtained in a manner consistent with the Declaration of Helsinki [34]. No one received compensation or was offered any incentive for participating in this study.

When participants in the baseline study could not be traced or were deceased, investigators were asked to fill in an ad hoc form reporting clinical information available at the last contact or, whenever possible, the cause of death. All baseline measures [32] were reported in previous studies and assessed at follow-up. Exclusion criteria and a detailed description of the study assessment procedures are previously reported [32, 35].

### Clinical evaluation

The Positive and Negative Syndrome Scale (PANSS) [36] was used to rate symptom severity. Disorganization was assessed using three items of the PANSS scale: P2 (conceptual disorganization), N5 (difficulty in abstract thinking), and G11 (poor attention). Positive symptoms were assessed using four items of the PANSS: P1 (delusions), P3 (hallucinatory behavior), P5 (grandiosity), and G9 (unusual thought content). We used the consensus five-factor solution proposed by Wallwork et al. [37].

Negative symptoms were assessed using the Brief Negative Symptom Scale (BNSS) [38], which includes five negative symptom domains: anhedonia, asociality, avolition, blunted affect, and alogia; for the purpose of the present study, as already done in our previous network analysis [39], we used two factors: “expressive deficit” (sum of the subscales blunted affect and alogia) and “avolition” (sum of the subscales anhedonia, asociality, and avolition). The Italian version of the scale was validated as part of the NIRP activities [40].

Depressive symptoms were evaluated using the Calgary Depression Scale for Schizophrenia (CDSS) [41].

Neurocognitive functions were rated using the Measurement and Treatment Research to Improve Cognition in Schizophrenia (MATRICS) Consensus Cognitive Battery (MCCB) [42, 43]. This battery includes tests for the assessment of seven cognitive domains: processing speed, attention/vigilance, working memory, verbal learning, visual learning, SC, and reasoning and problem solving.

The assessment of SC included the awareness of social inference test (TASIT) [44], which is a ToM test consisting of seven scales (positive emotions, negative emotions, sincere, simple sarcasm, paradoxical sarcasm, sarcasm enriched, and lie), organized into three sections: emotion recognition, social inference (minimal), and social inference (enriched). The assessment also included a test contained in the MCCB: the Mayer–Salovey–Caruso emotional intelligence test (MSCEIT) managing emotion section [45], which examines the regulation of emotions in oneself and in one’s relationships with others, and the facial emotion identification test (FEIT) [46], which examines emotion perception. Patient recovery at the 4-year follow-up was defined, consistent with Galderisi et al. [47], as the presence of symptomatic remission according to Andreasen et al. [48], and the presence of functional recovery, defined as a weighted score of at least 76.2 on SLOF “interpersonal relationships,” “work skills,” and “everyday life skills” scales.

### Statistical analysis

The reliable change index measures the extent to which the observed change in a scale from baseline to follow-up exceeds the one attributable to measurement error [49–51].

It is computed as follows:

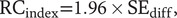

where 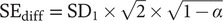 , 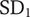 is the standard deviation of the scale at baseline and Cronbach’s α is the reliability of the scale.

We computed the reliable change index on the subset of patients who completed the SC scales at the two assessments (*N* = 548) and Cronbach’s α using data from baseline participants.

A clinically significant change occurs when a patient moves from the dysfunction in SC to “normal” SC. This requires the availability of data from a normative sample. In the NIRP study, normative data were obtained from healthy subjects recruited through flyers from the community at the same sites as the patient sample, using a stratified design by age, gender, and education within geographical macro-areas [52]. To determine the cut-off for clinically significant change at which the probability to belong to the dysfunctional or the functional population is the same, we used the following formula:

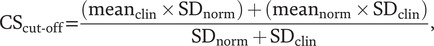

 where “clin” and “norm” stand for clinical and normative.

We then used a scatterplot to depict the follow-up scores versus the baseline scores.

Then, to denote RCSCs over time in SC, patients in the same group not exhibiting a reliable change were classified as “stable,” those moving to better or worse groups were classified as “improved” or “worsened”, respectively, and those exceeding the cut-off score for clinically significant change were classified as *clinically and significantly improved (CS).*

Stepwise multiple logistic regression models based on likelihood ratio statistics (entry criterion *p* = 0.05, removal criterion *p* = 0.10) were used to identify the predictors of reliable improvement and clinically significant improvement in SC (TASIT sect. 2 score).

NC variables and disorganization, in addition to baseline TASIT2 score, were included as potential predictors of these outcomes given their relationship with SC found in Mucci et al. [35].

Lastly, we investigated the effect of change in TASIT2 on recovery at follow-up after adjusting for age, gender, NC variables, and disorganization using a multiple logistic regression model.

Results are expressed as OR and 95% confidence interval.

## Results

Of the 921 participants recruited at baseline, 548 provided data on SC at both waves and were analyzed in the present study. The baseline patient characteristics are reported in Table 1.

**Table 1.** Demographic and clinical characteristics of study participants at baseline (*N* = 548).

Variables	
Gender (% males)	69.0
Age (mean ± SD)	40.4 ± 10.4
Years of education (mean ± SD)	11.9 ± 3.3
% Married	7.1
% Working	13.7
Age at first psychotic episode (mean ± SD)	24.1 ± 7.0
% Antipsychotic treatment	98.2
% First-generation antipsychotic	13.9
% Second-generation antipsychotic	70.6
% With both	13.7
% Integrated treatment	30.1
% Suicide attempts	17.9

### Changes in SC scores at 4 years

Statistically significant increases in SC scales were found for the simple and paradoxical sarcasm of TASIT scales, and for the total score of section 2, it includes these two scales. No change was detected for FEIT and MSCEIT scores and for the other two TASIT subscales (Table 2).

**Table 2.** Social cognition scale scores at baseline and follow-up.

Variable	Mean	SD	Variable	Mean	SD	*p*-value
TASIT_PE	8.75	2.218	TASIT_PE	8.82	2.299	0.485
TASIT_NE	11.60	3.185	TASIT_NE	11.79	3.069	0.110
TASIT_SI	15.27	4.893	TASIT_SI	14.91	4.080	0.076
TASIT_SS	11.84	4.867	TASIT_SS	12.35	4.655	**0.005**
TASIT_PS	11.14	4.725	TASIT_PS	11.67	4.620	**0.007**
TASIT_LI	19.92	5.737	TASIT_LI	20.13	5.447	0.366
TASIT_SA	18.70	6.505	TASIT_SA	18.81	5.886	0.603
TASIT section 1	20.35	4.849	TASIT section 1	20.61	4.795	0.137
TASIT section 2	38.05	10.721	TASIT section 2	38.93	10.560	**0.023**
TASIT section 3	38.61	10.982	TASIT section 3	38.94	10.071	0.407
MSCEIT	91.48	14.705	MSCEIT	90.53	14.729	0.138
FEIT	37.02	8.382	FEIT	37.61	8.315	0.672

FEIT, the Facial Emotion Identification Test; MSCEIT, the Mayer–Salovey–Caruso Emotional Intelligence Test; TASIT_LI, The Awareness of Social Inference Test, Lie; TASIT_NE, The Awareness of Social Inference Test, Negative Emotions; TASIT_PE, The Awareness of Social Inference Test, Positive Emotions; TASIT_PS, The Awareness of Social Inference Test, Paradoxical Sarcasm; TASIT_SA, The Awareness of Social Inference Test, Sarcasm Enriched; TASIT_SI, The Awareness of Social Inference Test, Sincere; TASIT_SS, The Awareness of Social Inference Test, Simple Sarcasm.

Bolded values stand for Statistically significant values (p < 0.05).

Therefore, to analyze changes in SC, we focused on the total score of TASIT sect. 2.

### Reliable change indices

The reliable change index was 9.8 (rounded-off to 10), indicating that a >10-point change is needed to state with 95% confidence that a real change has occurred in a patient and the clinically significant cut-off was 45, the mean and SD of the TASIT sect. 2 subscale for the normative population being 50.16 ± 8.02.

**Figure 1.**
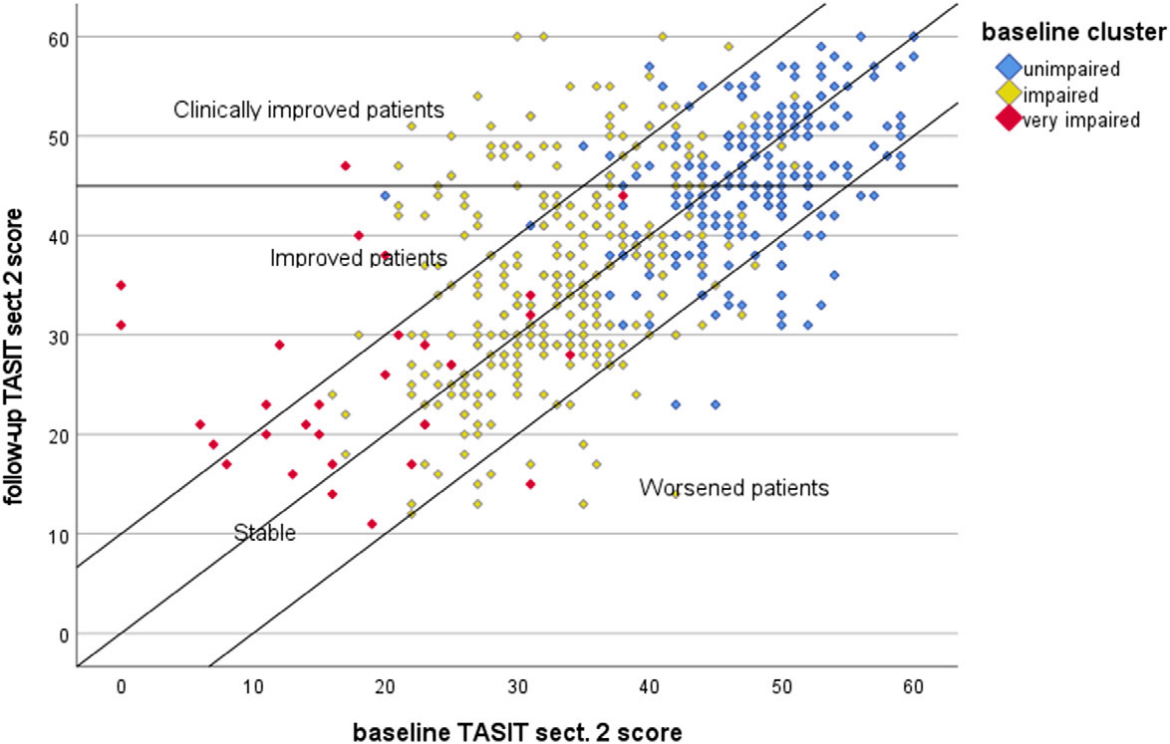
Scatterplot of baseline and follow-up scores of TASIT section 2. Markers denote the baseline patient cluster according to the algorithm developed by Rocca et al. [46] (unimpaired: TASIT simple sarcasm score > 13.5; impaired: TASIT simple sarcasm score ≤ 13.5 and TASIT lie score > 9.5; very impaired: TASIT simple sarcasm score ≤ 13.5 and TASIT lie score ≤ 9.5). TASIT sect. 2, The Awareness of Social Inference Test, section 2.

Figure 1 shows the scatterplot of TASIT sect. 2 scores for patients assessed at the two waves. The area delimited by the bars includes stable patients (*N* = 439, 80.1%), the area below the bars patients who worsened (*N* = 40, 7.3%) at follow-up and above the bars patients who improved (*N* = 69, 12.6%). Lastly, the area above the cut-off of 45 includes the subset of improved patients who achieved good SC (*N* = 44, 8%).

In a previous study, using baseline NIRP data [52], we developed a classification and regression tree algorithm to stratify patients by level of SC impairment using the ToM domains simple sarcasm and lie. In order to relate this stratification with the present findings, we classified patients according to the baseline cluster defined by the algorithm. Unimpaired patients were 228 (41.6%), impaired patients were 291 (53.1%), and very impaired patients were 29 (5.3%). Figure 1 indicates that the large majority of unimpaired and impaired patients remained stable, but a sizable proportion moved to the areas of clinical improvement and even one-third of the very impaired patients (9/29) scaled up and achieved better SC.

### Predictors of reliable change and clinically significant reliable change

In a forward stepwise multiple logistic regression analysis, only lower baseline TASIT2 score (OR = 0.940, 95% CI 0.922–0.959) predicted reliable improvement of TASIT sect. 2.

Similarly, in a second logistic regression analysis, only lower baseline TASIT2 scores (OR = 0.954, 95% CI 0.927–0.981) predicted a reliable and clinically significant improvement of TASIT sect. 2.

### Predictors of symptomatic and functional recovery

At the 4-year follow-up, 99 (18.1%) patients met the criteria for symptomatic and functional recovery. Of these, 31 already met recovery criteria at baseline and 68 achieved recovery at 4 years. Forty-one patients with baseline recovery no longer met the criteria at follow-up. We investigated whether the change in TASIT sect. 2 predicted functional recovery. Notably, after adjusting for age, gender, baseline NC variables, baseline recovery, and PANSS disorganization, change in TASIT sect. 2 scores predicted functional recovery (OR = 1.044, 95% CI 1.014–1.074; Table 3). In other words, because a 1-point change predicts a 4% increase in the probability of recovery, a 10-point change (corresponding to reliable improvement) predicts a 40% increase in the probability of recovery.

**Table 3.** Results of the multivariable logistic regression model predicting recovery at follow-up.

	*b*	SE (*b*)	*p*	OR	95% CI	
Age	−0.022	0.014	0.111	0.979	0.953	1.005
Gender	0.150	0.285	0.598	1.162	0.665	2.032
ΔTASIT2	0.043	0.015	**0.003**	1.044	1.014	1.074
PANSS disorganization	−0.144	0.044	**0.001**	0.866	0.795	0.944
BACS	0.017	0.016	0.279	1.017	0.986	1.049
Category fluency	−0.018	0.034	0.584	0.982	0.919	1.049
Processing speed	0.448	0.351	0.201	1.565	0.787	3.112
Working memory	−0.018	0.183	0.921	0.982	0.687	1.405
HVLT-R	0.063	0.031	**0.041**	1.065	1.002	1.132
NAB	0.016	0.024	0.494	1.016	0.970	1.065
Baseline remission	0.685	0.317	**0.031**	1.984	1.065	3.696
Constant	−1.243	1.551	0.423	0.288		

ΔTASIT2, change in The Awareness of Social Inference Test, section 2; BACS, Brief Assessment of Cognition in Schizophrenia; HVLT-R, Hopkins Verbal Learning Test-Revised; NAB, Neuropsychological Assessment Battery; PANSS disorganization, Positive and Negative Syndrome Scale disorganization.

Bolded values stand for Statistically significant values (p < 0.05)

## Discussion

The aim of this study was to estimate the magnitude and clinical significance of SC change in outpatients with stable SZ and to determine whether changes in SC over 4 years predicted patient recovery at follow-up.

To the best of our knowledge, this is the largest study carried out to follow-up SC, on two out of four SCOPE study’s SC domains, conducted on patients with stable SZ.

This study shows several key results relevant to clinical practice.

First, we found that the performance on tests of emotion processing (MSCEIT), emotional intelligence (FEIT), and some aspects of ToM (TASIT 1 -The Emotion Evaluation Test- that assesses the recognition of spontaneous emotional expression, such as being happy, surprised, sad, anxious, angry, disgusted, and neutral, and TASIT III, social inference enriched, that assesses lies versus sarcasm) were stable over 4 years, as shown by the absence of significant changes in mean scores from baseline.

Our results are in line with earlier findings showing longitudinal stability of SC test performance, also in patients in chronic phase [12, 20, 30] and support trait-like stability of SC in SZ. Previous studies of emotion processing in SZ reported the stability of performance over 6 and 12 months [29, 30]. Maat et al. [53] demonstrated that facial affect identification is significantly related to symptom severity rather than to a longer duration of illness, showing that patients who stay in remission for 3 years improve on emotion processing performance over time, whereas patients, who return to a non-remission state after 3 years, perform worse at follow-up as compared to baseline. Horan et al. [31], in 55 recent-onset SZ patients, showed both stability of emotion processing performance over 1-year follow-up, and a cross-lagged association between baseline SC scores and functional outcome 12 months later that supports a causal model in which baseline SC drove later functional outcome in the domain of work, above and beyond the contribution of symptoms. In the same way, Hoe et al. [20] demonstrated that baseline SC is shown to predict subsequent change in community functioning 12 months later in 130 outpatients with SZ. McCleery et al. [12] found that performance on tests of emotion processing and social perception was highly stable over a 5-year follow-up period in an outpatient sample of younger individuals with SZ.

Moreover, as our sample is composed of predominantly older, clinically stable individuals in chronic phases of SZ with a longer duration of illness than earlier studies, our findings are in contrast to the hypothesis that decline in SC performance occurs at a later illness stage of illness.

Patients in our study were not exposed to any specific and specialized intervention for SC over the 4-year follow-up. Thus, in the absence of tailored treatment, SC does not significantly change over a 4-year follow-up period. This stability of performance in the absence of a SC intervention does not address the question of whether SC impairments are amenable to training or treatment [12].

Indeed, SC training programs targeting multiple and specific core domains of SC have provided promising results in improving SC skills, which, in some cases, has translated into improvements in functional outcomes [54].

Second, statistically significant increases in SC scales were found for the simple and paradoxical sarcasm of TASIT scales, and for the total score of sect. 2, the social inference–minimal test, that includes these two scales and assesses comprehension of sincere versus sarcastic exchanges. ToM has been considered as the key process of SC, because it is connected to different abilities, such as social-perception, emotional processing, empathy, and social awareness [55].

As proof of this, in a previous study of our research group [52] the ToM domains were the most important for determining the SC clusters in SZ as compared with emotion perception and emotional intelligence: patients in the unimpaired cluster have a higher ability to grasp sarcasm than patients in the impaired and very impaired ones, whereas patients in the impaired cluster show a higher ability to understand lies than those in the very impaired cluster. Comprehension of sarcasm requires refined emotional skills such as empathic appreciation of the listener’s emotional state [56, 57], reflecting second-order mental representation and hierarchically higher-level SC ability.

In the present study, after 4 years, we found that even if the large majority of unimpaired and impaired patients remained stable, a sizeable proportion moved to the areas of clinical improvement and even one-third of the very impaired patients achieved better SC.

Our results are in contrast with previous evidence of more significant impairment in ToM performance with length of illness [58, 59] and in accordance with two longitudinal studies finding a ToM improvement over time [60–62], that can be related to symptoms.

The first one [60] showed that 14 patients in an acute exacerbation of SZ performed poorly on metaphor ToM tasks relative to a group of psychiatric controls before, but not after, remission. The second one [61] follows a sample of mostly 17 drug-free first-episode subjects (SZ 76%, schizoaffective disorder 6%, schizophreniform disorder 18%), over 6 weeks, after the beginning of antipsychotic medications, showing that both PANSS positive scores and ToM improved after medication was started, particularly during the first 2 weeks of antipsychotic treatment, but these changes were not associated, suggesting a dissimilar cognitive or neurobiological substrate for the two.

Three hypotheses have been proposed to explain the relationships between ToM and psychotic symptoms, that is, ToM as a mediator, or a moderator or co-occurring deficit [61]. According to the first one, impaired ToM could be a mediator of the formation and maintenance of psychosis, in which case ToM would be a causal factor of psychotic symptoms, and as some variables or treatment impact on ToM, this would then modify psychotic symptoms. According to the moderator hypothesis, the change in ToM would not directly correlate with a change in psychotic symptoms. According to the third hypothesis, ToM and psychosis are both downstream consequences of other illness variables and are not causally related to one another. Thus, baselines impairments of one do not predict change in the other; nor are the changes themselves associated with each other. The resolution of psychotic symptoms may be accompanied by improvements in ToM abilities but these improvements will not be associated.

It is unclear why only ToM improved over 4 years in our study, despite the absence of specialized interventions. No cognitive, demographic, and clinical characteristics that we have collected in our study predicted its improvement. Frith [62] has suggested that, in contrast to individuals with autism, ToM skills in patients with SZ develop normally but are “lost” following the psychotic episode. Thus, the remission of the acute episode may be accompanied by ToM improvement [63].

One possibility is that our original sample was closer to a psychotic episode at baseline and this clinical instability had a larger general impact on ToM deficits, “switching on” at the start of an acute episode and “switching off” at recovery. As patients continued to stabilize, a ToM improvement emerged by the 4-year follow-up. Nonetheless, these interpretations must be interpreted as generated, not confirmed, hypotheses.

Moreover, both the use of different psychometric scales and different statistical analyses may explain the divergent findings.

As for the psychometric scales, in most ToM studies, participants are asked to read short stories or cartoons and perform a first- or second-order mental attributions, which means inferring the mental state of a character in the story, or inferring the character’s beliefs about another character. We employed TASIT as an ecologically valid measure of simple (basic emotion perception) and complex (ToM skills) SC. The TASIT are visual ToM tasks, using videotaped conversational interactions (videoed scenes/animation), that closely align with real-world social encounters, contrary to the non-dynamic, cartoon-based ToM tasks. Sarcasm items are more psychometrically difficult, and sarcasm perception involves a more skillful and granular application of social inference that is likely to develop later than more blatant inferences used for detecting lies [56, 64–67].

As for the statistical analysis, traditional methods to evaluate SC changes in psychiatry, that is, percentage change in scores of a rating scale, effect size calculation, or improvement in terms of standard deviation from baseline, do not include normative data and therefore are not suitable to determine the extent to which a patient moves from dysfunction to “normal” SC. This simple and reliable method to define CS change in TASIT2 scores could be adopted in clinical care. This method allows the identification of individual’s outcome and could be used to monitor SC performance. The calculation of statistical significance and effect size at group level leads to an overestimate of effects, whereas calculation of CS changes in individual patients is a more conservative and meaningful way to assess outcome [68].

In the present study, we found that significant changes in TASIT2 were achieved only by 69 patients (12.6%), 44 of whom achieved good SC. Two reasons may explain this finding: one is the adoption of a conservative definition of SC improvement, and the second is a ceiling effect, as 41.6% of patients were in the unimpaired SC cluster at baseline, so the margin for SC improvement for the whole sample was modest.

Third, we sought to identify the cognitive, demographic, and clinical characteristics that predicted reliable improvement in TASIT2. Findings indicated that only lower TASIT baseline scores were significantly associated with TASIT2 reliable and clinically significant improvement. Thus, our results suggest that TASIT2 improvement is independent of symptom state.

Fourth, at the 4-year follow-up, 99 (18.1%) patients met the criteria for symptomatic and functional recovery. This finding is consistent with prior findings indicating that one in seven patients with SZ achieve a recovery phase that reflects both normalized social and vocational functioning and symptoms remission and lasts ≥2 years. Across studies, SC is considered one of the most important determinants of recovery in SZ, with both direct and indirect effects, mediating NC; however, it is unclear which SC domain most strongly affects recovery [69].

Our results suggest that the improvement in the ability to grasp simple and paradoxical sarcasm predicts recovery at 4 years. Indeed, as the attainment of reliable improvement in TASIT sect. 2 predicts a 40% increase in the probability of recovery in our study, this index measure has a clinically relevant meaning. To rule out the possibility that the relationship between TASIT2 and recovery was due to underlying neurocognitive impairment or other relevant symptoms [70], we adjusted our analyses for baseline NC variables and PANSS disorganization.

Deficits in SC may represent a substantial barrier for individuals with SZ in effectively responding to interpersonal conflict and constructing a meaningful account of the gains or losses experienced in life, leading to struggles in responding to adversity and making it possible to find meaning in life and to adapt to change on an ongoing basis [71–73]. Moreover, it may be that deficits in sarcasm detection impede social interaction and the establishment of peer-relationships, which can adversely affect real-life functioning to a great extent [74].

The strengths of the current study include its longitudinal design, a large and well-defined sample, the assessment of two SC domains, and the use of a sound statistical analysis.

However, our research has some limitations. First, the assessment battery only included measures of two of the four primary SCOPE domains of SC [2]; that is, emotion processing and ToM/mental state attribution. Second, the SZ sample included relatively stable outpatients and may not generalize to individuals with more severe symptoms or those receiving inpatient treatment. Third, we only assessed the participants twice across 4 years, which may have limited the possibility of detecting different trajectories of change.

## Conclusions

In summary, this longitudinal study indicates that SC improves reliably over time in about 1 in 10 patients. RCSC index provides a conservative way to assess SC variations and is associated with symptomatic and functional recovery at 4 years.

Clinically, a greater understanding of the role of ToM, that is, the developmental trajectories across a larger life span as well as potential predictors and moderators, may provide opportunities for targeted recovery-focused interventions. Growing evidence from treatment development research in chronically ill patients suggests that specific SC deficits can be improved through targeted skills training approaches, such as training programs that target ToM deficits.

## Data Availability

The data that support the findings of this study are available from the author.
